# Targeted Therapies and Druggable Genetic Anomalies in Acute Myeloid Leukemia: From Diagnostic Tools to Therapeutic Interventions

**DOI:** 10.3390/cancers13184698

**Published:** 2021-09-19

**Authors:** Francesco Lanza, Ali Bazarbachi

**Affiliations:** 1 Hematology Service and Romagna Transplant Network for HSCT, 48121 Ravenna, Italy; 2 Department of Internal Medicine, American University of Beirut Medical Center, Beirut 1107-2020, Lebanon

Acute myeloid leukemia (AML) is a clonal disorder resulting from acquired somatic mutations in hematopoietic progenitor cells that lead to the dysregulation of differentiation and the proliferation of hematopoietic cells [1,2]. There is accumulating evidence that a great number of genomic alterations, such as chromosomal rearrangements; gene amplifications, deletions, and mutations are essential for AML classification [1,2,3,4,5,6]. Furthermore, the identification of genetic lesions plays an increasing role in the prognosis and treatment of AML patients [1,2,3,4]. Next-generation sequencing (NGS) as well as whole-genome sequencing (WGS) has been recently integrated into clinical practice, allowing for a better risk stratification of AML patients. In fact, the routine use of NGS methodology has made possible the identification of one or more somatic mutations in more than 90% of patients with AML [1,2,3,4,5,6,7,8,9]. The most frequently mutated genes include *NPM1*, *FLT3*, *DNMT3A*, *IDH1*, *IDH2*, *TET2*, *RUNX1*, *TP53*, *WT1*, *NRAS*, and *CEBPα*. However, the prognostic-predictive significance of genetic mutations is more important in normal karyotype AML [4,10,11].

These aberrations might help to identify AML pathways of clonal dominance and shifts that could assist hematologists in targeting precision medicine therapies [7,8,9].

Over the last few years, there has been an increasing understanding of molecular aberrations that trigger the development of AML, and an increase in the use of novel molecular biology technologies, thus facilitating the development of investigational drugs targeting driver genetic mutations [7,8,9]. Based on this consideration, it could be postulated that identifying “druggable” mutations paves the way for the use of novel targeted therapies [12]. This Special Issue of *Cancers* focuses on the novel diagnostic and therapeutic tools for the management of AML with the main aim of improving our knowledge in the field of AML [10,11,12,13,14,15,16,17].

Twenty years ago, the discovery of imatinib for the treatment of chronic myeloid leukemia and its remarkable activity raised hopes for similar benefits in targeted therapies for AML. Over the last few years, precision medicine has been proposed for several hematological malignancies including acute leukemia, and more than 100 different targets have been identified in AML, making it an optimal candidate for experimental clinical studies [18,19,20,21,22].

Targeting fms, such as tyrosine kinase-3 (FLT-3), have been the first example of clinically actionable mutations, making them an attractive target for hematologists, as well as for pharmaceutical and biotechnology companies developing novel drugs [23]. Over the last few years, a great number of FLT-3-targeting drugs have been developed and tested in clinical trials [24,25]. The main consideration for the use of FLT-3 targeting compounds is related to the notion that the internal tandem duplications of the FLT3 gene (FLT3- ITD-mut) characterize a significant number (25–30%) of AML cases and represent an independent predictor of poor prognosis associated with an increased risk of relapse. In the Ratify trial, the addition of midostaurin to daunorubucine- and cytarabine- based induction therapy (the so-called “3 + 7”regimen) resulted in a significant improvement in patient outcome for the very first time [23,24,25]. More recent data have shown that FLT3 inhibitors produced better results in those patients carrying a specific insertion point of the ITD of FLT3 (located in the beta1-sheet region) compared to patients with other ITD insertion sites. Moreover, the use of midostaurin is associated with longer survival in patients with a high allelic FLT3-ITD ratio who lack NPM1 mutations (NPM1wt/FLT-3-ITDhigh). Interestingly, even after allogeneic stem cell transplantation (allo-SCT), FLT3-mutated AML is associated with a higher risk of early relapse (up to 60%) [26].Chemotherapy or FLT-3 inhibitors alone, or combined with donor lymphocyte infusions, show unsatisfactory long-term therapeutic effects. Therefore, several studies have investigated the use of post-transplant maintenance with FLT-3 inhibitors as a strategy to reduce the risk of relapse after allo-SCT. Some of these studies showed very promising results, making the use of FLT-3 inhibitors relatively popular in this clinical setting [26,27,28,29,30,31].

More recently, a great number of experimental drugs targeting single metabolic pathways have been developed and tested in phase 1, 2 and 3 clinical trials [12,17,18,19,20,21,22]. The question remains: what is the main reason for using these agents in AML?

Metabolism is a complex biochemical process that mammalian cells use to generate energy and maintain their growth and survival. Metabolism encompasses the generation of energy (ATP), the synthesis and breakdown of amino acids, glucose, fatty acids, and oxidative phosphorylation. In AML cells, metabolism can promote tumor growth and cellular proliferation. As a consequence, these alterations have emerged as a potential therapeutic target [21,22].

Some of these compounds proved to be effective in targeting altered metabolism, such as in the Krebs/citric acid cycle, hedgehog pathway (glasdegib), and the mitochondrial oxidative and carrier-associated secretory membrane proteins (so-called SCAMP), making the AML therapeutic armamentarium more flexible and intriguing. The identification of actionable bioenergetic cell pathways is rapidly expanding, allowing us to hypothesize new potential therapeutic modalities in AML [18].

More recently, the use of Bcl-2 inhibitors (venetoclax), and IDH inhibitors data have raised hopes for the benefits of targeted therapies in AML. For example, in older/unfit AML patients, hypomethylating agents (HMAs) and venetoclaxare now the new standard of care in this setting [12,13,14].

AML is also known to be an epigenetically heterogeneous disease. Based on this consideration, hypomethylating agents such as 5-azacitidine, decitabine, and guadecitabine; HDAC inhibitors (pracinostat, bromodomain and extraterminal (BET) family protein, among others), as well as novel agents targeting the aberrant activation of gene signaling (m-TOR-CDK9; syk inhibitors; Toll-like receptor signaling) have been tested in clinical trials.

Oncoprotein degradation is the basis of the curative effect of the combination of arsenic trioxide and all-trans retinoic acid in acute promyelocytic leukemia and has shown promising results in *NPM1*-mutated AML.

As far as immunotherapy is concerned, immunotherapy-based protocols have demonstrated modest activity in AML which needs to be confirmed in larger series. In contrast, encouraging results have been observed in acute lymphoblastic leukemia and in non-Hodgkin and Hodgkin lymphoma [12,13,14].

Interestingly, novel drugs and small compounds have been developed that can activate p53 in AML cells expressing the wild-type form of p53; the clinical implication related to the use of these molecules is under investigation [12].

A comprehensive list of druggable genetic lesions and targeted therapy in AML is shown in Table 1.

The role of minimal residual disease detection for the sharper management of acute leukemia is also considered in this issue, provided that the authors include a clinical setting in which one or multiple druggable genetic lesions are presented [3,8,31].

With this background, the journal *Cancers* is launching a collection of articles dealing with NGS data and the use of targeted therapies, as well as cellular therapies [32,33,34].

## Figures and Tables

**Table 1:** Druggable genetic lesions and targeted therapy in AML.

Number	Type of Genetic Lesion or Metabolic Pathways Which Can Be Used for Targeted Therapy
1	FLT3 mutation: FLT3 inhibitors (midostaurin, gilteritinib, crenolanib, sorafenib, quizartinib, etc.)
2	IDH1 and 2 mutation: IDH1/2 mutant inhibitors (ivosidenib, enasidenib, olutasidenib) – BCL2 inhibitors (venetoclax)
3	NPM1 mutation: BCL2 inhibitor (venetoclax); All-trans retinoic acid; Arsenic trioxide, Actinomycin D, Menin inhibitors.
4	KMT2A rearrangement: Menin inhibitors (SNDX-5613 and KO-539)
5	PIM 1-2-3 kinase inhibitors
6	Metabolic Pathway Targets: drugs targeting altered metabolism (such as Krebs/citric acid cycle); hedgehog pathway inhibitor (such as glasdegib); mitochondrial-targeted chemotherapeutics; SCAMP-carrier-associated secretory membrane proteins, and interfering drugs
7	BCL2 pathway: BCL2 inhibitors (venetoclax and new drug formulations); MCL1 inhibitors
8	Cell Surface Antigens: CD33 Antibody drug conjugate (gentuzumab ozogamicin, Vadastuximab talirine); anti-CD47 monoclonal antibody (Magrolimab); anti-CD70 monoclonal antibody (ARGX-110), and bi-specific antibodies such as anti-CD123 (flotetuzumab, etc.)
9	Epigenetic Pathway: Hypomethylating agents (5-azacitidine, decitabine, guadecitabine, CC-486), HDAC inhibitors (pracinostat, bromodomain and extraterminal (BET) family proteins; etc) and the targeting of aberrant activation of gene signaling (m-TOR-CDK9; syk inhibitor: entospletinib; Toll-like receptor signalling: CA-4948, small molecule inhibitor of IRAK4)
10	Immunotherapy: Wilms Tumor 1-WT1 target therapy (Galinpepimut-S); Checkpoint inhibitors targeting PD1/PDL1 (Nivolumab and pembrolizumab); CTLA4 (ipilimumab), and TIM3 inhibitor (sabatolimab)
11	Disruption of adhesion molecules and heparinoids: dociparstat, CX-01: CXCL12/CXCR4 axis), and uproleselan.
12	TP53 mutation: MDM2 inhibtors (such as Idasanutlin, miladematan, siremadlin, RG7112, etc.)
13	Cellular therapies: CAR-T, CAR-NK, and related CAR molecules
